# Diagnostic imaging of traumatic pseudoaneurysm of the thoracic aorta

**DOI:** 10.2478/v10019-010-0026-8

**Published:** 2010-09-09

**Authors:** Serif Beslic, Nermina Beslic, Selma Beslic, Amela Sofic, Muris Ibralic, Jasmina Karovic

**Affiliations:** Institute of Radiology, Sarajevo, Bosnia and Herzegovina

**Keywords:** traumatic pseudoaneurysm, chest radiography, CT, *i.v*. DSA, MRI

## Abstract

**Background:**

The purpose of the study was the presentation of findings and diagnostic imaging in patients with traumatic pseudoaneurysms of the thoracic aorta, as a rare consequence of road traffic accidents.

**Patients and methods:**

In 22 years we have found 8 traumatic pseudoaneurysms of the thoracic aorta, out of which 7 (87.5%) in male and 1 (12.5%) in female patients. At the time of accidents the youngest patient was 21 and the oldest was 55 (mean age 33.8 years), and at the moment of diagnosing a pseudoaneurysm they were 26 and 55 years old, respectively (mean age 38.7 years). In all patients chest radiography was performed as well as CT scan, in 6 (75%) patients intra-venous digital subtraction angiography was performed (*i.v.* DSA) and in 1 (12.5%) MRI. CT was performed with the application of 120 ml, and *i.v.* DSA with 60 ml of contrast medium, respectively.

**Results:**

In 8 (100%) patients, who suffered a road traffic accident, and whose chest radiograph showed the enlargement of the aortic knob and widening of the mediastinum, CT, *i.v.* DSA and MRI revealed a traumatic pseudoaneurysm of the thoracic aorta. Periods of time between the accidents and the initial diagnosis of the pseudoaneurysm varied from 7 days to 18 years (median 2.0 years). The diameter of the pseudoaneurysm was from 4.5 to 9.2 cm (median 5.5 cm). In 7 (87.5%) isthmus was involved, and in 1 (12.5%) descending thoracic aorta, respectively. The chest radiograph revealed marginal calcifications in 4 (50%), and on the CT in 5 (62.5%) patients. Intraluminal thrombosis was found by CT in 2(25%) traumatized patients.

**Conclusions:**

Traumatic pseudoaneurysm should be taken into consideration in blunt chest trauma, where a chest radiograph shows suspicious regions. A multislice CT is a diagnostic method of choice.

## Introduction

Back in 1557, Vesalius described a post mortem finding of an aortic rupture, which he suspected had been caused by a trauma. During the First World War, the aortic rupture was frequently noticed in victims of plane crashes. Immediately after the Second World War, in 1947, Strassman published 72 cases of aortic traumas with frequency of 1%.^1^ The development of traffic and motor vehicles generated more interest in traumatic changes, which became more and more frequent. In Ireland, the annual mortality caused by damage to thoracic blood vessels during motor vehicle accidents was approximately 3% for the period 1995 – 1998. In 1966, Greendyke reported about a number of victims of accidental deaths whose bodies had been examined in an autopsy, where 10% of whom had suffered from aortic rupture, mostly in motor traffic accidents. He stated that in one out of six fatal accidents, the victim suffered from the aortic rupture.^2^,^3^

Injuries to the aorta may be caused by a direct penetration of the aorta by a knife, bullet or a foreign body, or they can be a result of a blunt trauma.^4^ They can appear as incomplete aortic rupture, and as traumatic aortic dissection.^5^ Survivors may develop chronic traumatic aneurysm or pseudoaneurysm.^6^

Chronic post-traumatic thoracic aneurysm of aorta is a secondary dilatation in the rupture of the isthmus of aorta, which may be unnoticed in the moment of trauma.^7^ Most cases of aneurysm are located in the inner side of the aortic arch with a ventral extension.^8^ In contrast to atherosclerotic aneurysm, post-traumatic aneurysm occurs in younger people, where 90% patients are younger than 45. In almost 50% of cases, they were detected accidentally during a systematic radiographic examination. A detailed medical history can assist in associating this aneurysmatic formation with a trauma which had previously occurred many years ago.^7^ The traumatic aortic rupture may be difficult to be diagnosed, and if missed, it almost always leads to a fatal result. Thoracic radiography is considered to be a very useful screening method.

## Patients and methods

During the period of 22 years we have found 8 post-traumatic pseudoaneurysms of thoracic aorta, in 7 (87.5%) male and 1 (12.5%) female patient. At the moment of their accident the youngest patient was 21 and the oldest was 55 (mean age 33.8), and at the moment of diagnosing a pseudoaneurysm they were 27 and 55 years old (mean age 38.7). Over the period of 22 years different diagnostic methods were used, depending on technological achievements and diagnostic tools that we have had on disposal at different times. All 8 (100%) patients had a chest radiograph and CT scan performed (7 examinations were performed on sequential Somatom DR, and 1 on the four-row Volume Zoom Siemens), 6 (75%) patients had *i.v*. DSA and 1 (12.5%) patient had a MRI scan. CT examinations were performed with the *i.v*. application of 120 ml, and *i.v*. DSA with 60 ml of contrast medium. MRI scan examination was performed on Magnet 1T Siemens unit, with the “time of flight” (TOF) sequence. Considering that 7 examinations were performed on sequential CT unit, and only one on the multislice machine, the “road maps” review was acquired with *i.v*. DSA in 6 cases and once with MRI, before making a decision concerning the surgical treatment.

## Results

**FIGURE 1A, B:**
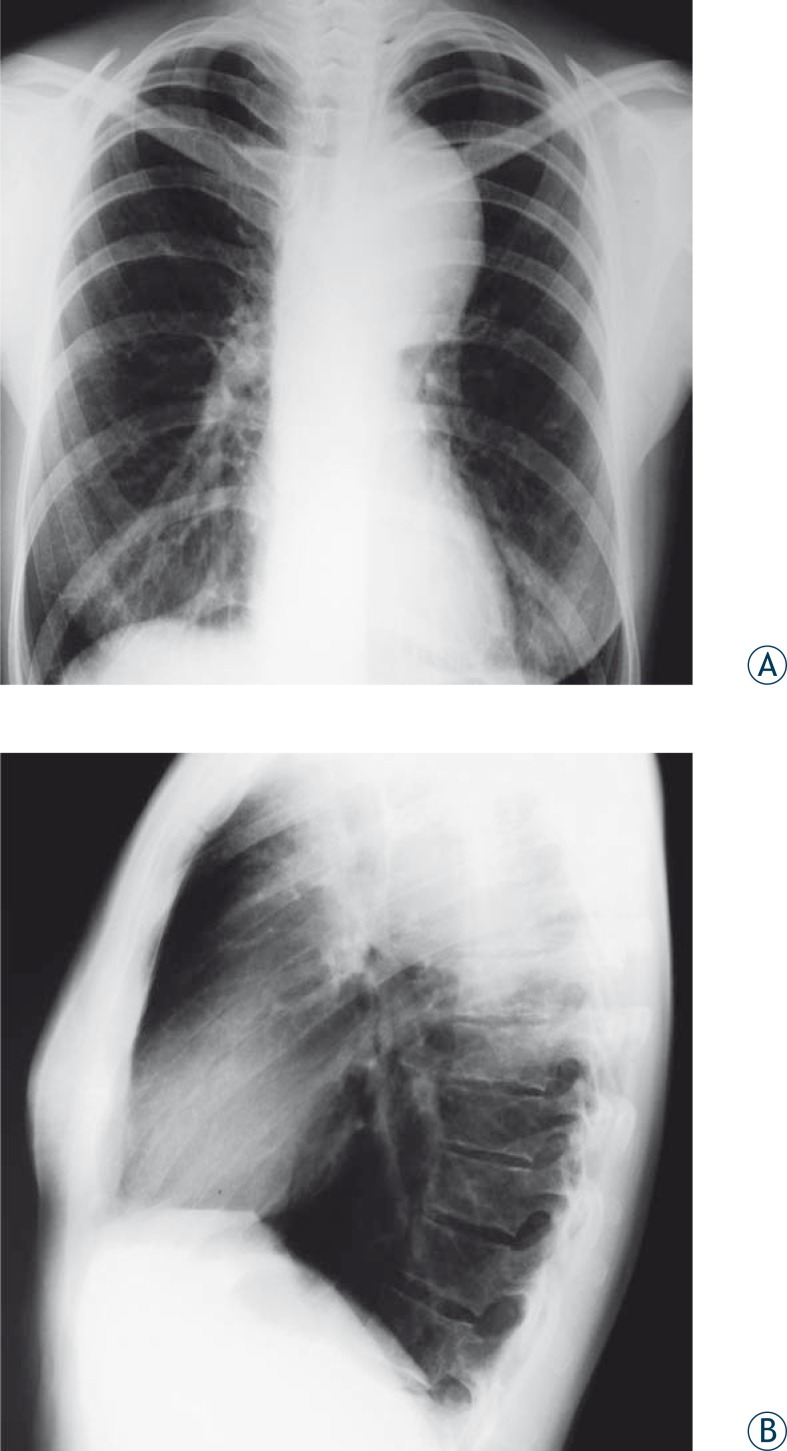
Chest x – ray of aortic knob enlargement.

**FIGURE 2A, B:**
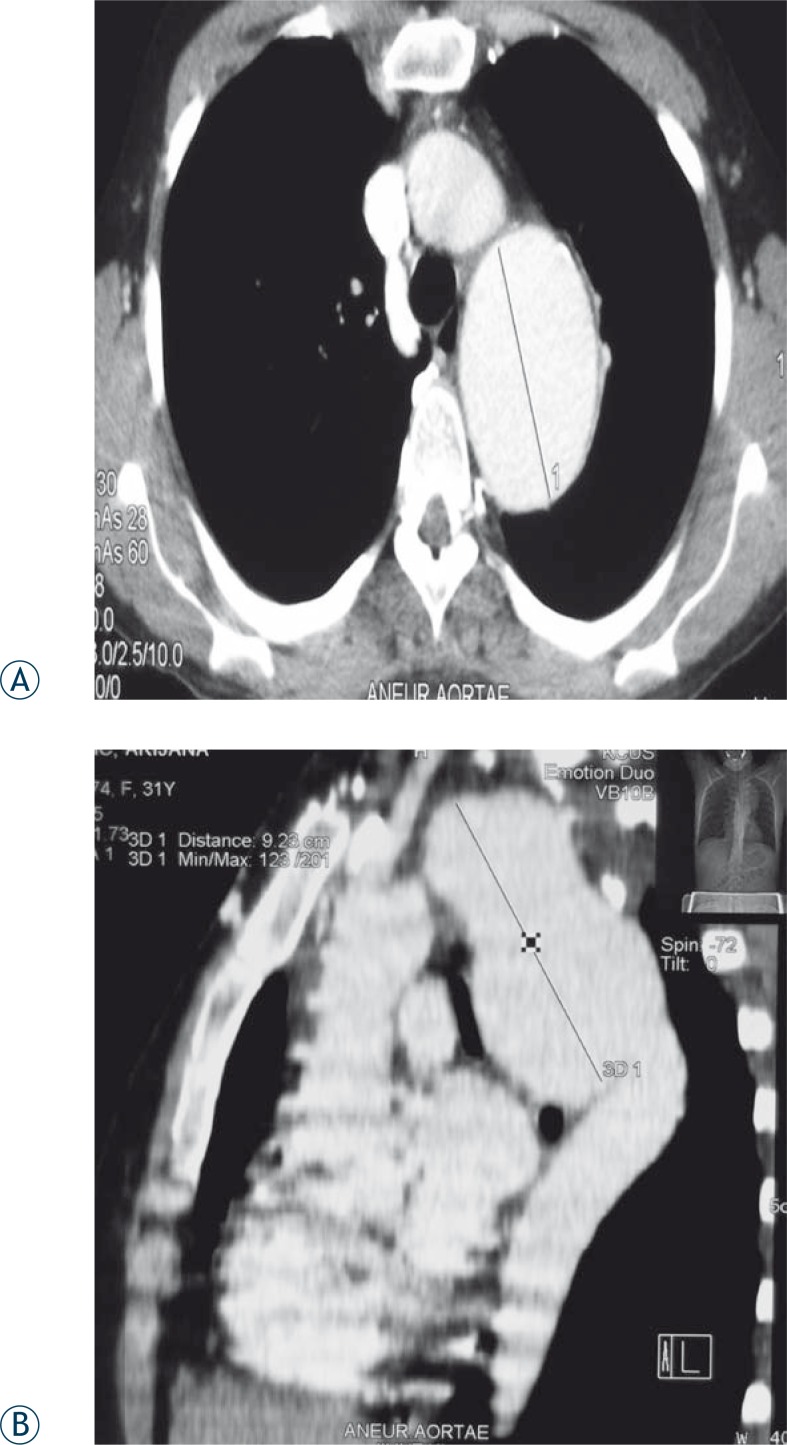
CT of posttraumatic pseudoaneurysm of the aortic isthmus.

**FIGURE 3:**
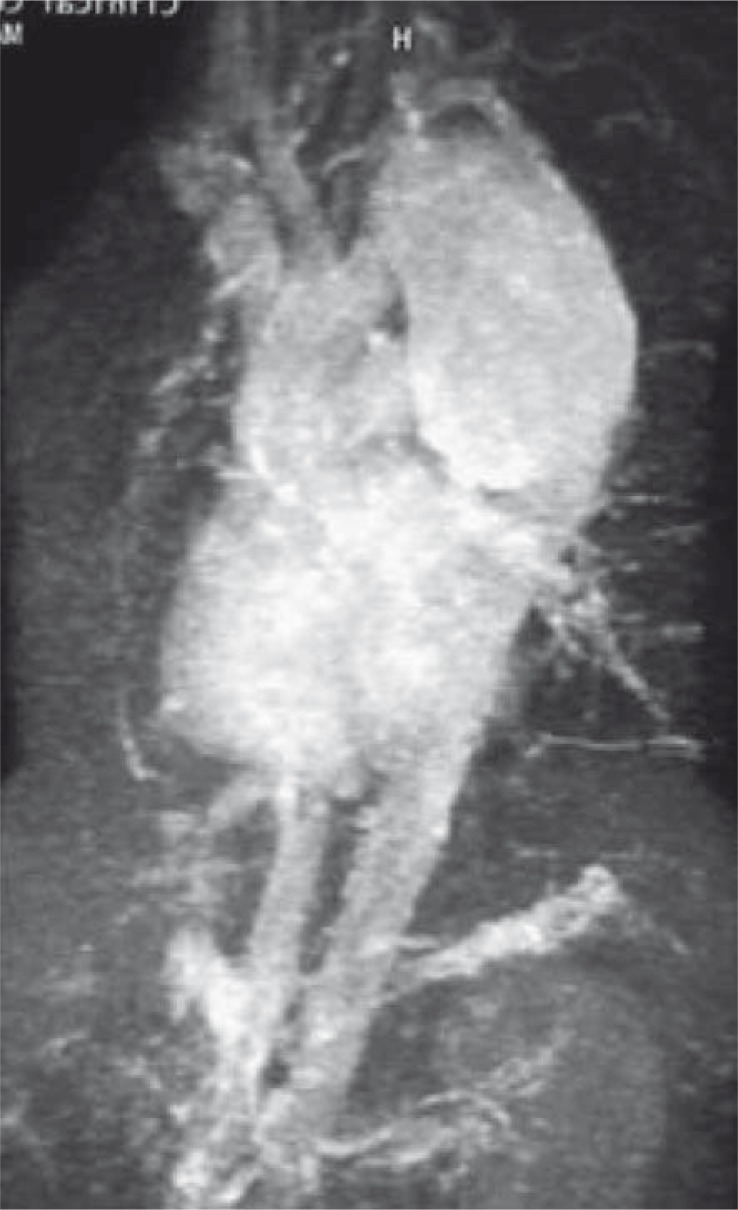
TOF MRI of posttraumatic pseudoaneurysm of the aortic isthmus.

In 8 (100%) patients who suffered road traffic accidents, who had signs of mediastinal widening and enlargement of the aortic knob (Figure 1A, B), some had lamellar calcifications as well, the traumatic pseudoaneurysm was proved by CT (Figure 2A, B), *i.v*. DSA and MRI (Figure 3). In 7 (87.5%) patients it was a chronic posttraumatic pseudoaneurysm, and in only one case it was an acute post-traumatic pseudoaneurysm. The period from the accident to the diagnosis of the pseudoaneurysm was 7 days in the last patient, and in the others it was between 2 months and 18 years (median 2.0 years). The diameter of the pseudoaneurysm was 4.5 to 9.2 cm (median 5.5 cm). In 7 (87.5%) injured patients the isthmus was involved, and in 1 (12.5%) case it was the descending thoracic aorta. In all 8 (100%) patients the chest radiograph has shown the enlargement of the aortic knob and widening of the mediastinum. The chest radiographs have shown marginal calcifications in 4 (50%), and CT in 5 (62.5%) patients. Intraluminal thrombosis was discovered by CT examinations in 2 (25%) traumatized patients. All three methods have shown the presence of the posttraumatic pseudoaneurysm of the thoracic aorta. The chest radiograph was the initial method indicating pathology (Figure 1A, B). The largest diameter of the posttraumatic pseudoaneurysm was 9.2 cm, and it was detected in the pregnant patient 8 years after the accident, while the aneurysm detected 18 years after the accident in a male patient had a diameter of 7 cm. The characteristics and localization of the posttraumatic pseudoaneurysms are presented in Table 1.

CT scanning had a 100% specificity and sensitivity, and also provided the additional information on the wall calcification and thrombotic masses, as well as some other findings in the thorax.

Six (75%) patients were treated with the surgical placement of a Dacron graft, and the last case diagnosed with multislice CT 7 days after the injury, was treated with the placement of a stent graft. One patient did not agree to undergo a surgical operation, and has been occasionally checked up for a longer period.

## Discussion

The traumatic rupture of the thoracic aorta is a rare condition in critically injured victims of a blunt trauma. The cause for the trauma are falls from heights >3 m, and motor vehicle crashes, so that some authors believe that all victims of significant decelerating traumas should be referred to an angiographic examination of the aorta.

A blunt trauma can damage the thoracic aorta with several mechanisms, by the fracture dislocating thoracic vertebrae, or by the penetration of the first rib and clavicula. A very high pressure may occur in the aorta due to various forces occurring in the thorax caused by acceleration, either in horizontal or vertical plane in the moment of impact (the effect of a water hammer) and the rupture is caused by an explosive burst.

During a motor traffic accident, the descending aorta remains fixed to the back thoracic wall by means of interosseous arteries, whilst the heart and ascending aorta contort toward the front and the split occurs within the isthmus, which is the most common rupture site.^1^

According to the referential data, about 90% of injuries include the region of the aortic isthmus, immediately distal from ligamentum arteriosum, and the left subclavical artery with ripping of vessels of the arch. Splits of ascending aorta, of distal descending aorta, or of abdominal aorta are much less common.^7^,^9^ In this analysis we had 7 (87.5%) isthmic lesions and one lesion of the descending thoracic aorta.

According to the literature, most victims die immediately (85%), whilst those who manage to get to hospital, if they are properly diagnosed, may have a surgical or another reparation procedure done. The aortic injury may be limited to a partial circumferential split in the intima and/or media of the aortic wall.^5^ About 15–20% of them survive an acute episode long enough to manage to get to hospital, with a periaortic haematoma and false aneurysm since adventitia has not been ruptured yet.^3^,^4^,^10^

The traumatic rupture of the thoracic aorta may occur at any time, sometimes during the examination procedure, whilst some patients appear to be in a more stable condition. Many patients can be referred to treatments of other life threatening conditions before the reparation of the traumatic rupture of the thoracic aorta, although a postponed therapy is not routinely recommended.^11^

According to some data, about 2% to 5% of patients are discharged from hospital without being diagnosed with the traumatic pseudoaneurysm of the thoracic aorta, so that a chronic post-traumatic aneurysm develops later on.^4^,^7^,^8^ In most cases, they suffer a secondary rupture, which results in death later on. However, there are rare reports in medical literature about a prolonged period of surviving.^3^

It has been suggested in medical literature that the enlargement and the risk of the rupture progressively increase the longer the aneurysm is present.^8^ In this analysis, posttraumatic pseudoaneurysm of the aorta was found in 8 patients, out of which in 7 (87.5%) cases at the isthmus, which is in accordance with the literature.

The median time period from the accident until the detection of pseudoaneurysm was 2,0 years, while in one patient it was as long as 18 years. This demonstrates the potentially long survival period in some patients.

The primary damage of the aortic wall probably influences the development and the size of the aneurysm. With time, lamellar calcifications will develop around the pseudoaneurysm, which were found in 62.5% of cases, mainly in patients with the pseudoaneurysms over 2 years old. Calcifications can be seen in a chest radiograph, and especially on multislice CT. It is possible that they have a certain role in the length of the survival in such patients.

Intramural thrombosis in these patients was rare, in only 2 (25%) patients. All described post-traumatic pseudoaneurysms were chronic, except in one patient where it was detected 7 days after the accident.

The traumatic rupture of the thoracic aorta is a highly lethal condition and is often combined with multiple injuries, all of which may require an immediate evaluation and treatment.^11^

Clinicians keep perplexed how such a catastrophic lesion presents with so few real symptoms and signs. It is crucial that the emergency health practitioner recognizes it in making an initial diagnosis. Quite often those subtle changes remain unnoticed in the initial stage and remain undetected until the complete rupture and death occur.^3^

Symptoms of the traumatic rupture of the thoracic aorta include: chest pain, dyspnoea, backache, harsh voice, dysphasia and cough, as well as contusion of the front wall of the chest, with the impression of the wheel on the front part of the chest, and the acute coarctation syndrome.^3^,^9^ The unstable condition of a bleeding patient should also raise suspicion.

Thoracic radiography is an initial analysis most frequently used for verifying the traumatic rupture of the thoracic aorta. It is indicative in almost half of all cases. The interpretation of thoracic radiography in the patient in lying position is difficult. The extension and changes in the shape of the mediastinum on radiography are the most important signs of the aortic rupture and traumatic pseudoaneurysm of the aorta (Figure 1A, B). The most frequently radiographic signs reported are: abnormal shape of the aorta, shading of aortic-pulmonary window, left major bronchus shift downwards, deviation of trachea to the right from the medial line, shift of the nasogastric tubus, apical cap and border calcifications on the outer part of the mass, etc. Haematomas most commonly come from small arteries and veins in the mediastinum. Some 7.3% of patients have a normal mediastinum presented on the initial radiography, unless the traumatic pseudoaneurysm has been followed by a mediastinal haemorrhage, or haematoma, or the pseudoaneurysm has been very small, or it has been located in such manner as to keep the mediastinal shape unchanged.^3^,^8^

In this study, the chest radiography in 8 patients (100%) with post-traumatic pseudoaneurysm of the thoracic aorta, showed an enlargement in the mediastinum, as well as an enlargement of the aortal knob. In 4 patients (50%), lamellar border calcifications were also noticed. History of the road traffic accident directed the diagnostic method.

Until recently, aortography was the most accepted standard amongst methods of screenings for the signs of the traumatic rupture of the thoracic aorta. The problem with aortography is that it is an invasive method which uses iodine as a contrast medium, and it also gives an inadequate picture of intraluminal thrombosis. Although aortography was the most accepted standard, it was not free from false-positive and false-negative results. The problem is also the transportation of a potentially unstable patient to the area for the vascular examination.

In this analysis, the posttraumatic pseudoaneurysm was in 7 (87.5%) cases confirmed with sequential CT scan and in 6 (75%) cases it was supplemented with *i.v*. DSA. In 1 (12.5%) case the posttraumatic pseudoaneurysm was confirmed with MRI, and the last one with the multislice CT (Figure 2A, B).

In 1976, transoesophageal echocardiography appeared, as a method to complement and possibly substitute aortography of the arch in the evaluation of unstable multiple traumatized patients with a potential rupture of the aorta. The examination is less invasive, it does not require a contrast medium, it can be performed with the patient lying in bed, and lasts for 15 minutes. However, it depends highly on the professional who performs it, it is not always available, and there is also a problem due to securing airways and cervical spine.

With the emergence of a spiral CT (SCT), it has confirmed to be efficient for screening of critically injured patients with the traumatic rupture of the thoracic aorta. In 1998, Wicky *et al.* reported that with the blunt trauma to the chest and injury to the aorta, CT has 100% of sensitivity, and 99.8% of specificity, with 89% positive and 100% negative predictive values, and the total diagnostic accuracy of 99.7%.^12^ CT examination results typically consist of a sack-shape bulge which has been demarked from the aortal lumen with a collar. According to this author, an operation based on CT is safe and expeditious. A positive SCT leads to thoracotomy, whereas a negative one excludes it. According to this author, an angiogram is unnecessary and it only delays a definite therapy, whilst a positive CT is the only diagnostic method which needs to be done prior to referring a patient to the theatre.^10^

Contrary to the conventional angiography, a multilayered CT angiography not only shows blood vessels, but it also allows an evaluation of nearby structures, or determining of optimal stent-graft values.^13^ The endovascular stent graft, is a very useful method in managing the aneurism as a minimally invasive procedure.^14^

A more liberal use of CT in evaluation of patients with a blunt trauma has resulted in making more diagnoses of aortal splits, which could certainly be treated without surgery.^13^ The diagnosis of post-traumatic pseudoaneurysm in this study has been confirmed by using CT in 8 (100% cases), *i.v.* DSA in 6 cases (75%), and MRI in 1 case (12.5%). CT has confirmed to be an excellent method in depicting the aneurismal sack, calcifications and thrombus within the pseudoaneurysm (Figure 2A, B).

A cardiovascular MRI is a non-invasive examination, without irradiation, for the evaluation of the real and false aneurysm of aorta. 3D gadolinium-enriched magnet resonance angiography collects information and thus enables a detailed examination of aneurysm and its relationship with other structures.^6^ The weakness of this procedure is in its slowness, also its frequent inability to connect to monitoring devices needed in urgent situations, and a poor visibility of calcified deposits and changes in pulmonary parenchyma (Figure 3).

Until recently the treatment of patients with the traumatic injury of the thoracic aorta has been surgical with an operative death rate between 3.5–4.6%, or the risk of a post-operative paraplegia.^8^,^9^,^15^ However, the latest studies emphasise that patients treated with the endovascular stent graft stay shorter in hospital compared to those who have been referred to the open surgery.^15^

In this study, 6 patients were treated with the open surgery and the placement of a Dacron graft, 1 patient was treated with the placement of an endovascular stent graft, while one patient has rejected an open surgery and has been periodically checked up.

## Conclusions

It is significant to point out the hidden nature of the traumatic rupture of the thoracic aorta, which may be undiagnosed and many years later accidentally revealed as a chronic posttraumatic pseudo aneurysm of the thoracic aorta, unless it ruptures in the meantime and threatens the patient’s life.

Pseudoaneurysm should be considered in patients with a blunt chest trauma, and with suspicious chest radiographic findings. Multislice SCT angiography is a fast, safe and non-invasive imaging technique which can prevent the occurrence of late pseudoaneurysm formation of the thoracic aorta, with an excellent depiction of the aneurysmatic sack, marginal calcifications and thrombotic masses within the pseudoaneurysm, as well as of associated thoracic lesions. All departments that receive trauma patients should have a multislice CT. In most trauma centres CT screening is an integral part of the diagnosis and care of patients with serious blunt injuries.

## Figures and Tables

**TABLE 1:** Characteristics and localization of the posttraumatic pseudoaneurysms

**No.**	**Sex**	**Period from trauma to diagnosis**	**Diameter of the aneurysm**	**Calcifications**	**Thrombotic masses**	**Involved segment of the aorta**	**Treatment**
1	M	7 days	4.8 cm	−	−	Isthmus	Stent
2	M	2 years	5.2 cm	+	−	Isthmus	Dacron graft
3	M	60 days	4.5 cm	−	−	Isthmus	Dacron graft
4	M	18 years	7.0 cm	+	+	Isthmus	Dacron graft
5	M	2 years	5.5 cm	+	+	Descending aorta	Dacron graft
6	M	9 years	6.0 cm	+	−	Isthmus	Refused treatment
7	M	180 days	5.5 cm	−	−	Isthmus	Dacron graft
8	F	8 years	9.2 cm	+	−	Isthmus	Dacron graft

## References

[1] Strassman G (1947). Traumatic rupture of the aorta. Am Heart J.

[2] Greendyke RM (1966). Traumatic rupture of aorta; special reference to automobile accidents. JAMA.

[3] O’Conor CE (2004). Diagnosing traumatic rupture of the thoracic aorta in the emergency department. Emerg Med J.

[4] Sutton D, Gregson RHS, Sutton D (2003). Arteriography and interventional angiography. Radiology.

[5] Alkadhi H, Wildermuth S, Desbiolles L, Schertler T, Crook D, Marincek B (2004). Vascular emergencies of the thorax after blunt and iatrogenic trauma: multi-detector row CT and three-dimensional imaging. Radiographics.

[6] Chai P, Mohiaddin R (2005). Traumatic pseudoaneurysm of the descending thoracic aorta. Circulation.

[7] Roques X (1991). Chronic post-traumatic aneurysms of the thoracic aorta. [French]. Rev Prat.

[8] Heystraten FM, Rosenbusch G, Kingma LM, Lacquet LK (1986). Chronic posttraumatic aneurysm of the thoracic aorta: surgically correctable occult threat. AJR Am J Roentgenol.

[9] Rogers FB (2004). Traumatic laceration of the aorta. N Engl J Med.

[10] Fraser RS, Colman N, Muller NL, Pare PD (2005). Synopsis of diseases of the chest.

[11] Downing SW, Sperling JS, Mirvis SE, Cardarelli MG, Gilbert TB, Scalea TM (2001). Experience with spiral computed tomography as the sole diagnostic method for traumatic aortic rupture. Ann Thorac Surg.

[12] Wicky S, Capasso P, Meuli R, Fischer A, von Segesser L, Schnyder P (1998). Spiral CT aortography: an efficient technique for the diagnosis of traumatic aortic injury. Eur Radiol.

[13] Gjikolli B, Hadzihasanovic B, Jaganjac S, Herceglija E, Niksic M, Hadzimehmedagic A (2008). Treatment of complicated case with subclavia steal syndrome and stenosis of common iliac artery. Radiol Oncol.

[14] Pavlisa G, Ozretic D, Rados M, Pavlisa G (2009). Migration of Enterprise stent in treatment of intracranial aneurysms: a report of two cases. Radiol Oncol.

[15] Lebl DR, Dicker RA, Spain DA, Brundage SI (2006). Dramatic shift in the primary management of traumatic thoracic aortic rupture. Arch Surg.

